# Important considerations in the derivation of background at sediment sites

**DOI:** 10.1002/ieam.4124

**Published:** 2019-03-04

**Authors:** Allison Geiselbrecht, Shahrokh Rouhani, Karen Thorbjornsen, Douglas Blue, Steven Nadeau, Tessa Gardner‐Brown, Steven Brown

**Affiliations:** ^1^ Floyd|Snider Seattle Washington USA; ^2^ NewFields Atlanta Georgia USA; ^3^ APTIM Knoxville Tennessee USA; ^4^ Imperial Oil Environmental Services Calgary Alberta Canada; ^5^ Honigman Miller Schwartz and Cohn LLP Detroit Michigan USA; ^6^ Dow, Environmental Remediation & Restoration Midland Michigan USA

**Keywords:** Contaminated sediment sites, Background concentration, Sediment geochemical evaluations, Outlier evaluation, Statistical population comparison

## Abstract

In the United States, there is an absence of federal guidance related to deriving and applying background concentrations at contaminated sediment sites. This absence has resulted in significant variability, uncertainty, and disagreement regarding how representative background concentrations of chemicals of concern should be derived for these sites. The present article discusses important considerations in the derivation of representative background concentrations to be used in the evaluation of contaminated sediment sites. Specifically, a thorough understanding of a site is critical to selecting the background reference areas from which representative background concentrations can be derived, representative background concentrations should account for contributions from those background chemical inputs (natural and anthropogenic sources) that will continue affecting the site even after remediation, perceived outliers should not be eliminated from the background data set just because they are the highest or lowest values, and geochemical evaluation of trace metals is a useful tool for deriving representative background concentrations. On a site‐specific level, representative background concentrations are critical for putting site‐related risk into context, developing a cost‐effective and technically feasible remedial approach, understanding the potential for recontamination, and ensuring long‐term remedy success. In a broader context, clear guidance from the United State Environmental Protection Agency (USEPA) for deriving and applying background concentrations for contaminated sediment sites would help promote national consistency in site assessment and remedy decision making. *Integr Environ Assess Manag* 2019;00:000–000. © 2019 The Authors. *Integrated Environmental Assessment and Management* Published by Wiley Periodicals, Inc. on behalf of Society of Environmental Toxicology & Chemistry (SETAC)

## Key Points

- Background reference areas should be as similar as possible to the site in terms of surrounding land use and with respect to physical, chemical, geological, and biological characteristics; but importantly, they should not be influenced by site releases.
- Representative background concentrations should account for input from natural and anthropogenic background sources that will continue to affect the site, allowing for defensible representative background concentrations to be used in remedial investigation, remedy selection, and monitoring.
- An outlier should not be eliminated just because it is the highest or lowest value in a data set; when the nature of a perceived outlier is unknown or disputed, specific and rigorous statistical analyses should be conducted to evaluate the effect of retaining or eliminating the data point.
- Once derived, representative background concentrations should remain fixed during remedial investigation, remedial response, and performance monitoring; otherwise, the lack of certainty for stakeholders will be deemed unreasonable and an impediment to the selection and implementation of any remedy.

## INTRODUCTION

The United States Environmental Protection Agency (USEPA) has recognized for more than 25 years that establishing a reliable representation of background is a critical issue at Superfund sites (USEPA 1989a). Although USEPA has provided guidance for establishing background chemical concentrations in soil (USEPA 2002), similar guidance is lacking for sediment sites. The present article details the results of the Background Workshop, held on November 15‐16, 2016, in Washington, DC, to outline key considerations in the development of representative background concentrations (refer to *Definitions* section) at sediment sites. Workshop attendees included representatives from industry, consulting, academia, and regulatory agencies (state and federal). The contents of the present article represent the consensus of most of the participants and should not be construed as representing the individual views of all attendees.

Clear direction for deriving representative background concentrations at sediment sites is needed because technically defensible, representative background concentrations are critical for putting risk into context; developing an appropriate, cost‐effective, and technically feasible remedial approach; understanding the potential for recontamination; and ensuring long‐term remedy success. Moreover, clear guidance from USEPA would help promote national consistency in site assessment and remedy decision making. The present article has been prepared to present the results of the multistakeholder technical workshop described earlier. As a fundamental premise, representative background concentrations should account for contributions from natural sources and nonsite‐related anthropogenic sources (i.e., contributions not related to a site release) that will continue affecting the site, even after remediation. Once established, representative background concentrations may be used to develop cleanup goals at sediment sites where background concentrations are greater than risk‐based cleanup levels. This concept is affirmed in USEPA guidance for determining background chemical concentrations in soils as follows: “The reasons for this approach include cost‐effectiveness, technical practicability, and the potential for recontamination of remediated areas by surrounding areas with elevated background concentrations” (USEPA 2002). The USEPA's approach for soil highlights the importance of deriving representative background concentrations that reflect actual background. In some cases, derived representative background concentrations may become de facto cleanup goals (i.e., where these concentrations are greater than risk‐based cleanup levels), thereby influencing the scope and scale of the remedy. Under these conditions, representative background concentrations help to define achievable remedial goals.

### Definitions

The following definitions, based on USEPA definitions, are used in the present article:

#### Background

Substances or locations that are not influenced by the releases from a site and are usually described as naturally occurring or anthropogenic (USEPA 1989a, 2002).

- Natural background. Naturally occurring substances present in the environment in forms that have not been influenced by human activity (USEPA 2002).
- Anthropogenic background. Natural and human‐made substances present in the environment as a result of human activities, not specifically related to the site‐related release in question (USEPA 2002).

#### Background reference areas

The areas where background samples for chemical concentrations are collected for comparison with samples collected on site. The background reference areas should have similar physical, chemical, geological, and biological characteristics as the site being investigated but should not have been affected by activities on the site (USEPA 2002). Although in many cases the background reference areas are situated off site, nonimpacted portions of the onsite areas may also be suitable as background reference areas (USEPA 2002). Consistent with USEPA guidance, the background reference areas should include anthropogenic inputs unrelated to the site that are reflective of the larger region.

#### Conceptual site model (CSM)

A representation of the environmental system and the physical, chemical, biological, and anthropogenic processes that determine the transport of contaminants from sources to receptors. Essential elements of a CSM generally include information about contaminant sources, transport pathways, exposure pathways, and receptors. A good CSM can be a valuable tool in evaluating the potential effectiveness of remedial alternatives (USEPA 2005).

#### Outliers

Measurements that are very large or small relative to the rest of the data and are suspected of misrepresenting the population from which they were collected.

- False outliers. Measurements that are very large or small relative to the rest of the data but represent true extreme values of a distribution and indicate more variability in the population than was expected (USEPA 2006).
- True outliers. Measurements that are very large or small relative to the rest of the data but are a result of transcription errors, data‐coding errors, or measurement system problems (USEPA 2006).

Additionally, the term “representative background concentrations” is used frequently throughout the present article. Representative background concentration is defined (for the purpose of this article) as a chemical concentration that is inclusive of naturally occurring and anthropogenic sources affecting the site but is not related to the site release in question. It is derived from sampling within representative background reference areas that may be located on site and/or off site but are not affected by a site release or site activities. For man‐made chemicals, the anthropogenic background concentration and the representative background concentrations are equivalent. For naturally occurring chemicals (e.g., metals), representative background concentrations are equivalent to the sum of the anthropogenic and natural background concentrations.

### Objectives for determining representative background concentrations and remedy decision making

At many sediment sites, multiple sources may contribute to the nature and extent of contamination. The largest contribution of contamination at Superfund sites is typically attributed to site releases. However, some contamination can also result from natural and offsite sources.

Contamination derived from off site (i.e., not associated with site releases) is considered a component of representative background concentrations and will continue to be a source of contamination to the site, unless all transport pathways are controlled. A primary objective of determining representative background concentrations is to account for any background chemical input (both natural and anthropogenic) that is expected to continue affecting the site. One of the guiding principles for management of contaminated sediment sites is that sources should be controlled to the greatest extent feasible prior to initiating remediation at the subject site. According to USEPA, “Generally, significant continuing upland sources… should be controlled to the greatest extent possible before sediment cleanup” (USEPA 2005). However, it is rarely feasible to control all background sources.

When representative background concentrations accurately reflect ongoing chemical inputs to a site from all sources, this results in defensible representative background concentrations for use in the remedial investigation, remedy selection processes, and performance monitoring. In addition to informing or establishing cleanup levels, representative background concentrations can assist in the following:

- Determining a site boundary
- Determining chemicals of concern
- Establishing a realistic long‐term monitoring plan, or optimizing existing long‐term monitoring plans
- Assessing remedy success.

In the absence of representative background concentrations for remedy decision making, risk‐based cleanup levels may be used inappropriately at sites where representative background concentrations are actually greater than risk‐based concentrations. Alternatively, if the representative background concentration has been underestimated or erroneously calculated, inappropriately low cleanup goals could be used in the remedy selection process. Inevitably, in both cases, these sites will eventually return to background conditions after the remedy has been completed, so the remedy would be considered a failure if the site did not meet cleanup goals over the short or long term. This has been demonstrated on a number of sediment sites throughout the United States, under both federal and state lead (Nadeau and Skaggs 2015). Moreover, attempting to clean up to concentrations less than actual background is not sustainable over the long‐term, can lead to unnecessary additional ecological disruption of sites, and can require considerable site remediation expenditures that serve no environmental or public health purpose. In some cases, if the representative background concentrations have been overestimated or erroneously calculated, inappropriately high cleanup goals could be used in the remedy selection process. This could result in the selection of a suboptimal remedy that is less protective of human health and the environment. The considerations discussed in the present article are intended to help promote a scientifically sound approach for establishing representative background concentrations, leading to decision making that avoids costly perceived remedy failures due to recontamination.

### Current regulations and guidance

At the federal level, background is discussed in a number of USEPA documents, but technical guidance describing protocols to derive representative background concentrations at sediment sites (as opposed to soil and groundwater at upland sites) has not been issued. The present article has been prepared in the absence of existing USEPA‐issued guidance on the derivation of representative background concentrations for contaminated sediment sites.

There are a number of relevant documents with information on the derivation of background concentrations for upland sites, including risk assessment and soil screening guidance (USEPA 1989a, 1989b, 1991, 1994a, 1994b, 1996, 1997, 2001, 2003, 2009), determination of background concentrations of inorganics in soils and sediments (Breckenridge and Crockett 1995), and characterization guidance of background chemicals in soil at Superfund sites (USEPA 2001).

Further, USEPA issued a guidance document in 2002 entitled “Role of Background in the CERCLA Cleanup Program” that seeks to clarify the “preferred approach for the consideration of background constituent concentrations of hazardous pollutants, and contaminants in certain steps of the remedy selection process, such as risk assessment and risk management” (USEPA 2002). That document is intended to serve as national policy and is the most current federal guidance on deriving and applying background at upland sites; it also finalizes the discussion of sampling and statistical analysis of representative background concentrations at soil sites. The 2002 USEPA guidance does not address sediment sites; the document indicates that “guidance may be updated in the future to address non‐soil media. Non‐soil media are dynamic and influenced by upstream or upgradient sources. Such media—air, groundwater, surface water, and sediments—typically require additional analyses of release and transport, involve more complex spatial and temporal sampling strategies, and require different ways of combining and analyzing data.” But considerations for sediment site characterization, as well as developing appropriate cleanup goals, are discussed in USEPA's 2005 guidance for remediation of contaminated sediment sites (USEPA 2005). However, the 2005 guidance does not provide a detailed discussion describing the derivation of representative background concentrations. Other federal documents include the guidance prepared by the US Department of the Navy (USDON 2003), which does discuss background analyses at sediment sites.

In addition to the federal guidance, some states have also issued guidance related to the derivation of representative background and/or the use of background; the present article focuses on representative background as it applies to federally regulated sites. State guidance is typically similar to federal guidance but may use different terminology or may vary in other ways, such as specific statistical procedures recommended for the screening of background data, characterization of background distributions, and calculation of background threshold values (BTVs).

Finally, in order to determine representative background concentrations, it is typically necessary to identify background reference areas. A separate, but related, concept that is not addressed here is the use of reference areas in ecological risk assessments. This involves identifying one or more suitable reference areas to facilitate sampling for the comparison of toxicological responses and/or resident biological communities (e.g., benthic macroinvertebrates). Note that there are additional documents relevant to the ecological risk evaluation process (e.g., USEPA 1997, 1999), and again, the terminology may be slightly different.

## ELEMENTS OF A CONCEPTUAL SITE MODEL

Representative background concentrations are typically derived from data collected from background reference areas. Selection of appropriate background reference areas depends on a thorough understanding of the site. As discussed, background reference areas should have key similarities to the site, reflecting similar physical, chemical, geological, biological, and land use conditions; and importantly, they should not be influenced by site releases. Although it is recognized that there will always be differences between the site and the background reference areas, they should be as similar as possible in these attributes.

A CSM is typically developed with the objective of obtaining and presenting a detailed understanding of a site, in order to select a background reference area. It may also provide an increased understanding of the factors that may contribute to the representative background concentrations. A CSM is “a representation of the environmental system and the physical, chemical, and biological processes that determine the transport of contaminants from sources to receptors” (USEPA 2005). The CSM should provide a robust understanding of the physical characteristics of the site, as well as the sources of contamination, potentially contaminated media, chemical transport pathways, and exposure pathways applicable for ecological and human receptors. The CSM is an important tool for selecting background reference areas, but it also provides additional clarity and steering for proponents, consultants, and the community, and it can highlight options for risk reduction.

Factors that typically contribute to representative background concentrations are summarized in the list below and are discussed in more detail in the Supplemental Data associated with this article. These factors should be considered when developing the study design for representative background determination.

- Land use. Contaminant concentrations tend to increase as the degree of urbanization increases (Kemble et al. 2013). The degree of urbanization positively correlates to the level of chemical input that can be expected to migrate onto the site before, during, and after the completion of the remedy. Thus, this should be considered in selecting background reference areas and determining representative background concentrations.
- Shoreline conditions. Shoreline conditions should be evaluated as part of CSM development (USEPA 2005) when screening and selecting background reference areas. A number of examples of recontamination in Superfund sites due to contaminated soil erosion (e.g., slumping under docks and scouring after high‐flow storm events) are described by the Association of State and Territorial Solid Waste Management Officials in “Sediment Remedy Effectiveness and Recontamination: Selected Case Studies” (ASTSWMO 2013).
- Urban runoff. Urban runoff is considered to be a significant contributor of contamination to watersheds and sediments and contains many chemicals most commonly found at sediment sites, such as polychlorinated biphenyls (PCBs), polycyclic aromatic hydrocarbons (PAHs), and metals (USEPA 1995). Chemical inputs from urban runoff to a site and background reference areas should be as similar as possible in order to obtain representative background concentrations for use at the site.
- Direct discharges. Chemical loading from wastewater and/or stormwater discharge is regulated under Clean Water Act programs, which may set limits for chemical concentrations for discharges from these conveyance systems but does not completely eliminate chemical loading from the direct discharge. Therefore, chemical loading to a site from direct discharges should be accounted for in representative background concentrations because they may continue to be a major source of chemical input, even after site remediation (ASTSWMO 2013).
- Sediment transport. Sediment sites are consistently receiving suspended sediments from offsite areas that contain background concentrations of contaminants from anthropogenic sources and may also contain concentrations of naturally occurring chemicals similar to the chemicals of concern for the site. Because sediment resuspension is a transport pathway for contamination, it is important to consider that representative background levels of contamination will inevitably move into a site through this natural process.
- Atmospheric deposition. Atmospheric deposition from industrial and urban areas, and areas near major transportation corridors, is a recognized pathway of contamination, particularly for those contaminants ubiquitously found in the environment, such as metals, PAHs, PCBs, and pesticides (USGS 2005; Amodio et al. 2014).
- Source control. Source control should be fully completed, or at least substantially completed, before remediation of a sediment site begins. If source control has not been completed or is not feasible, it is critical that the potential inputs from uncontrolled ongoing sources be included in the determination of representative background concentrations.
- Sediment physical properties. The physical properties of sediment strongly influence the distribution of naturally occurring and anthropogenic background chemicals in the environment. The abundance of organic material, inorganic material, and porewater within a sediment body; sediment texture; and sorption capacity should be evaluated with geotechnical testing and general chemistry analyses.
- Hydrodynamic environment and sediment profile. The vertical profile of sediment, rates of sediment deposition, erosion and removal, and mixing vary widely among aquatic environments and should be assessed as part of the CSM because these factors affect chemical distribution in sediments.
- Geochemistry. Metals concentrations in sediment are controlled by several factors, such as redox, pH, and adsorption–desorption reactions on mineral surfaces. These processes should be evaluated using analytical data for metals (major elements as well as trace elements of concern), anions (e.g., sulfide), and total organic carbon (TOC). When site and background metals concentrations are geochemically evaluated, then mechanistic explanations for elevated concentrations (i.e., perceived outliers) can be provided.

## CONSIDERATIONS IN DATA REVIEW AND EVALUATION FOR THE DETERMINATION OF BACKGROUND

Determination of background conditions at a sediment site almost always requires additional sampling and/or data analyses. To ensure the reliability of these evaluations, appropriate procedures should be considered during each phase of the investigation. Topics that require special attention include those related to the practical aspects of sampling design, selecting the representative background reference areas, using existing site data, choosing appropriate statistical methods for comparison, addressing outliers, and geochemical evaluation of sample data. These topics are discussed further in this section.

### Study design considerations

In general, unless existing contemporaneous data are adequate for extracting site‐specific background data (USDON 2003), additional sampling focused on the determination of representative background concentrations is necessary. This process is often initiated by identifying suitable background reference areas. All samples collected within the background reference areas should be considered representative of background. Typical components of a sampling design, including the selected type of samples, sampling depth, and sampling methodology for the background reference areas, should match those used during site data collection. The number and location of background samples can be determined based on a number of different statistical approaches, such as those provided by the US Department of Energy's Visual Sample Plan (VSP Development Team 2019) software tool.

Agency agreement on the scope and scale of the sampling effort to determine representative background concentrations is important and should be captured in a site's data quality objectives, using USEPA's Data Quality Assessment approach (USEPA 2006).

### Selection of representative background reference areas

One of the critical steps in a background analysis is the selection of representative background reference areas. As discussed, representative background reference areas should have “the same physical, chemical, geological, and biological characteristics as the site being investigated, but [have] not been affected by activities at the site,” and they should be informed by the CSM (USEPA 2002). Further, “the ideal background reference area would have the same distribution of concentrations of the chemicals of concern as those which would be expected on the site if the site had never been impacted” (USEPA 2002). Background reference areas need to include sources of contaminants that reflect the land use in the vicinity of the site, except for the inputs from releases or activities at the site. It can be advantageous to utilize multiple background reference areas (or subareas) when developing representative background concentrations.

Defining the extent of site influence in urban areas is very difficult and should be considered carefully. Selection of such an analogous area is complicated, due to the fact that sediment background often represents mixtures of naturally occurring and anthropogenic influences. In some cases, these mixtures yield geographically distinct background populations (i.e., background reference subareas with varying degrees of anthropogenic influences in different parts of the background reference areas). Under such situations, the parts of the targeted background reference areas (or subareas) that are most analogous to the site should be selected as the background reference areas. Selection of analogous background reference subareas is often supported by multiple lines of evidence, including degrees of urbanization, presence or absence of combined sewer overflows, prevailing sediment TOC content, and grain sizes.

### Use of existing site data

In many instances, site data include samples that are free of site influences. Particularly within a larger site data set, there will be samples not affected by site releases that will be reflective of representative background conditions. In these cases, statistical methods, such as probability plot analyses, are recommended for extracting site‐specific background data sets from existing site data sets (USDON 2003). This approach involves preparing iterative probability plots to determine break points, indicating a separation between the data points displaying site release impacts and the data points free of site release influence that are suitable for use in deriving representative background concentrations. This procedure is especially useful for extracting representative background concentrations from large site data sets (Geiselbrecht et al. 2015).

The extraction of representative background concentrations from site data not only maximizes the utility of existing data, but also avoids the often complex task of selecting separate background reference areas that adequately represent the site. Even when data from separate offsite background reference areas are available, an extracted site‐specific background data set provides an additional line of evidence for determining representative background concentrations. Therefore, an analysis of existing site data is always recommended.

### Statistical comparisons

Due to the different types of contamination (e.g., localized vs widespread), USEPA guidance recommends simultaneous use of multiple tests for a valid and complete comparison of background and site distributions (USEPA 2006). There are generally 2 broad statistical approaches for comparing site and background populations: 1) point‐by‐point comparisons and 2) background–site population comparisons.

The point‐by‐point comparison approach is based on comparing individual site measurements to a given BTV, either to delineate the extent of impact or to identify localized (or “hot spot”) contamination. A BTV is a specific value intended to define an upper limit to background concentrations for a given site. Common candidates for BTV include the upper tolerance limit (UTL; typically 95% with 95% coverage), the upper prediction limit (UPL; typically 95% confidence), and the upper simultaneous limit (USL; typically 95% confidence; USEPA 2005). Regardless of the chosen BTV, point‐by‐point comparisons are prone to produce excessive false‐positive errors. That is, as the number of comparisons increases, the chances of incorrectly concluding above‐background exceedances approach 100%, even when the site data are derived from the background population (Gibbons 1994). In other words, the odds are very high (approaching 100%) that the analysis would report exceedances of background, even when the results are truly within background ranges. In fact, the US Department of the Navy recommends against point‐by‐point comparisons, except when coupled with reverification sampling (USDON 2003).

**Figure 1:**
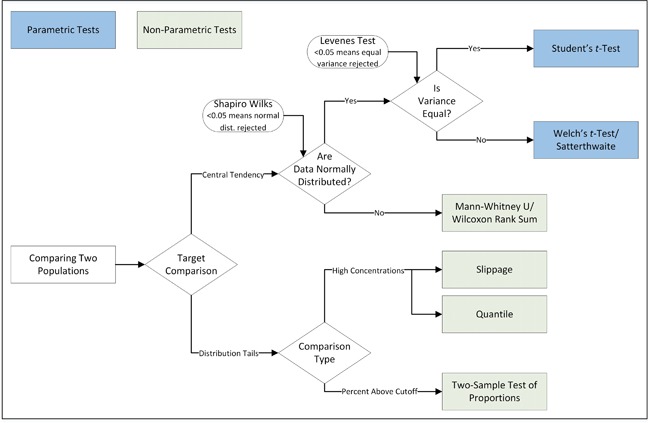
Statistical tests for comparison of 2 populations (adapted from USDON 2003).

The background versus site population comparisons are based on specific statistical hypothesis tests, as displayed on Figure 1. Some of these tests, such as the parametric *t*‐test and the nonparametric Mann–Whitney U test, are geared toward the comparison of central tendencies of 2 populations, to identify widespread contamination. Other tests focus on the comparison of the upper tails of the 2 populations to identify localized contamination. In many instances, both widespread contamination and localized contamination should be tested concurrently. Selection of the appropriate test is contingent on the specific conditions, including the target statistics of interest and the type of the distributions displayed by the investigated site and background data sets, as well as their variance equivalency. These tests are designed to maintain the false negative error rates at the user‐specified levels, often set at 1%, 5%, or 10%. In practice, nonparametric tests are often preferred because they do not require any specific distributional assumption about the investigated site and background data. Compared to point‐by‐point comparison, background–site population comparisons are less prone to excessive false‐positive errors.

### Outlier evaluation

Some background measurements may be perceived as outliers, which are measurements that are disproportionately large or small relative to the rest of the data, so they are suspected of misrepresenting the population from which they were collected (USEPA 2006). Outliers have been attributed to 2 broad categories of causes: 1) Outliers may represent very high or low values from the investigated population that have occurred by chance, or 2) outliers may be the results of errors such as faulty sample collection, laboratory equipment failure, and improper data entry (Grubbs 1969; USEPA 2002, 2006). Concentrations belonging to separate background subpopulations are also false outliers.

There are well‐established procedures in statistics to identify outliers, including visual inspection of graphs using particular techniques, such as probability and box‐and‐whisker plots, as well as statistical tests, such as Rosner's test and Dixon's test (USEPA 2002, 2006). The confirmation of true outliers, however, requires a thorough evaluation of the causes of such measurements to ensure that purported outliers are not improperly excluded, thereby skewing the statistical analysis. Grubbs's “Procedures for Detecting Outlying Observations in Samples” (Grubbs 1969) states:

“An outlying observation, or ‘outlier,’ is one that appears to deviate markedly from other members of the sample in which it occurs. In this connection, the following two alternatives are of interest: (1) An outlying observation may be merely an extreme manifestation of the random variability inherent in the data. If this is true, the values should be retained and processed in the same manner as the other observations in the sample. (2) On the other hand, an outlying observation may be the result of gross deviation from prescribed experimental procedure or an error in calculating or recording the numerical value. In such cases, it may be desirable to institute an investigation to ascertain the reason for the aberrant value. The observation may even eventually be rejected as a result of the investigation, though not necessarily so. At any rate, in subsequent data analysis the outlier or outliers will be recognized as probably being from a different population than that of the sample values.”

The USEPA has recognized the importance of properly evaluating apparent outliers and not excluding data points simply based on their magnitude. USEPA's “Data Quality Assessment: Statistical Methods for Practitioners” (USEPA 2006) divides outliers into 2 groups: 1) “true outliers” resulting from transcription errors, data‐coding errors, or measurement system problems such as instrument breakdown; and 2) “false outliers” representing true extreme values of a distribution (for instance, hot spots) and indicating more variability in the population than expected. This guidance states that “failure to remove true outliers or the removal of false outliers both lead to a distortion of estimates of population parameters.” In the data review, it is imperative that all sample data, including false outliers, are retained and are not arbitrarily removed. A proper statistical outlier evaluation will include (at least) the following steps, as discussed in USEPA's 2002 guidance:

- A careful investigation or review should be conducted for each statistical outlier, with scientific reasoning to ascertain the cause of the aberrant value (Grubbs 1969). If there is any error in collecting, transporting, or analyzing the sample or transcribing the data, then the error should be corrected.
- If the error cannot be corrected, the associated true statistical outliers should be eliminated from the background data set. (“Data points that are flagged as outliers should be eliminated from the data set if field or laboratory records indicate that the sample location was not a reasonable reference area, or if there was a problem in collecting or analyzing the sample.” [USEPA 2002]).
- If no error can be identified or confirmed, false outliers should not be arbitrarily eliminated.

Thus, an outlier should not be eliminated from the background data set just because it is the highest or lowest value in the data set or based on the perception that the outlying value is too high or too low to fit into the background data set. In this case, the outliers “may be merely an extreme manifestation of the random variability inherent in the data [and] the values should be retained and processed in the same manner as the other observations in the sample” (Grubbs 1969). True outliers should be deleted from data sets, and false outliers should be retained. In cases where the nature of the outliers is either unknown or disputed, all statistical analyses should be conducted with both the full and truncated data sets to evaluate the effect of retaining or eliminating the disputed outliers (USEPA 2002, 2006). In cases involving actual or potential true outliers, their removal is required before a valid BTV can be calculated. In such instances, a statistically rigorous method must be used for outlier identification and removal.

As noted by USEPA, it is critical that the considerations outlined in this discussion on outliers are followed during the statistical analyses of the background data. Exercising caution to not improperly exclude “false” outliers, which accurately represent conditions at the background reference areas, will ensure technically defensible derivation of representative background concentrations and will also avoid errors in the statistical approach when relying on a preconceived notion that outliers “distort statistics if used in any calculations” (USEPA 2002). Finally, once derived, representative background concentrations should remain fixed for the duration of the remedial investigation and remedial response. Otherwise, the lack of certainty for stakeholders would be an impediment to the implementation of any remedy.

### Geochemical evaluation of metals concentrations

Geochemical evaluation is a tool used to evaluate elemental (i.e., metals) concentrations in a given data set, which may include exceedances of representative background concentrations (Myers and Thorbjornsen 2004; Thorbjornsen and Myers 2007a). Consideration of geochemistry in the evaluation of trace metals concentrations in sediments does not require background reference area data for comparison, so, advantageously, it can be used when it is not otherwise possible to identify background reference areas. However, geochemical evaluation is more convincing when data from the background reference areas are available for inclusion in the evaluation.

Geochemical evaluation can be used to determine whether trace metal concentration values identified as outliers by statistical methods are actually the result of a release from the site or are simply manifestations of the geochemical variability in the site data set. When properly performed, geochemical evaluation provides mechanistic explanations for elevated concentrations (Myers and Thorbjornsen 2004; Thorbjornsen and Myers 2007a). It is important to keep in mind that geochemical evaluation is not a simple graphical technique; all potential geochemical mechanisms, field observations, and available data need to be considered when examining element concentrations. Useful sources of additional information on the geochemical mechanisms controlling metals concentrations in sediments include Windom et al. (1989), Balistrieri and Chao (1990), Stumm and Morgan (1996), Post (1999), Cornell and Schwertmann (2003), Keimowitz et al. (2005), and Kabata‐Pendias (2011).

Background data can be evaluated by using the ratios of specific element pairs that are based on the known geochemical behavior of trace elements and their association with specific sediment minerals. The USEPA's Target Analyte List of 23 metals (USEPA 2015) includes all of the common trace elements of interest, as well as the major elements that are used as reference elements.

Scatter plots may be prepared in which the concentration of a trace element of interest is plotted on the *y*‐axis and the concentration of a reference element, which represents the mineral (or organic compound) to which the trace element is adsorbed, is plotted on the *x*‐axis. For further analysis, a ratio plot may also be prepared; like the scatter plot, the concentration of the trace element of interest is plotted on the *y*‐axis, but the corresponding elemental ratio (trace element concentration divided by reference element concentration) is plotted on the *x*‐axis. If a metal is found at an elevated concentration and that sample's elemental ratio lies outside the range of background elemental ratios, then that sample should be examined further. For example, the elevated ratio might reflect anthropogenic input of the trace element from the site, or it may indicate that the trace element concentration of that sample is controlled by another geochemical process, such as reducing conditions or trace metal precipitation in the sediment. If the sample's ratio lies within the range of background elemental ratios, then it is considered representative of background conditions.

The selection of a reference element for the scatter or ratio plot should be based on a careful comparison of the reference element and the trace element of interest, as well as consideration of site‐specific geochemical processes. The following points provide a general overview of a few relevant elemental associations, but the scientific literature provides additional information.

- Clay minerals in the pH range of 6 to 8 have a strongly net negative surface charge and attract positively charged trace metal ions, so these trace metals adsorb to clay mineral surfaces. Aluminum is a primary component of all clay minerals, and detected Al concentrations in sediment serve as proxy indicators of the relative amounts of clay minerals (Myers and Thorbjornsen 2004; Thorbjornsen and Myers 2007a). In addition, Al concentrations are generally not influenced by chemical releases, and the element is not redox‐active. For these reasons, the concentrations of positively charged trace metals (such as Cu, Pb, Ni, and Zn) commonly covary with Al concentrations in uncontaminated sediment samples.
- Iron oxides (including hydroxides, oxyhydroxides, hydrous oxides, and amorphous oxides) typically have a net positive surface charge in the pH range of 6 to 8. Detected Fe concentrations serve as proxy indicators of the relative amounts of Fe oxide minerals in sediment samples from oxic environments (Myers and Thorbjornsen 2004). Due to their net positive surface charge, Fe oxides have an affinity for adsorption of negatively charged oxyanions (including As, Sb, Se, and V), so that the concentrations of these trace metals commonly covary with Fe concentration in uncontaminated samples from oxic sediments.
- Because metal species may be positively, neutrally, or negatively charged, other associations occur outside of these generalizations. Reference elements other than Fe and Al (typically Mn, which is a proxy indicator for Mn oxide minerals in oxic sediments) can also be used. Grain size and TOC content are additional reference parameters. For example, due to the affinity Hg has to adsorb onto organic matter, covariance of Hg versus TOC concentrations may be observed in the absence of site‐related Hg contamination.

Although quantitative statistical techniques are commonly applied to identify outliers or to develop pass–fail criteria for the presence of contamination, they are not recommended for geochemical evaluations that use scatter plots or ratio plots, for many scientific reasons (Thorbjornsen and Myers 2007b). For example, each trace element has varying degrees of correlation with the major elements with which it is associated; some trace elements have strong affinities for a particular mineral, whereas other elements will partition themselves among several minerals. Correlation coefficients, confidence limits, and prediction limits are highly influenced in a nonlinear manner by outliers, as well as by the analytical uncertainty associated with estimated concentrations less than the reporting limit. Evaluation of geochemical data can be quite complex because the effects of redox, pH, and other processes need to be considered. Trace‐versus‐major‐element correlations are usually not linear and often possess some degree of curvature; this also translates to a higher range of elemental ratios and greater spread of the samples along the *x*‐axis of a ratio plot.

Geochemical evaluation is an important line of evidence when evaluating background data and is commonly performed in conjunction with statistical evaluation of the data set. A properly performed geochemical evaluation examines the interrelationships between elements, in the context of all available data, for the purpose of identifying the processes controlling the observed concentrations. Scatter plots and ratio plots, coupled with knowledge of the geochemical behavior of elements in the site‐specific environment, may indicate that elevated concentrations that would otherwise fail statistical outlier tests have a natural and/or anthropogenic source that is not related to a site release. If the trace‐versus‐major‐element ratio lies within the ratio range of the representative background samples, then site‐related contamination is not indicated.

## CONCLUSIONS AND RECOMMENDATIONS

Derivation of representative background concentrations is critical to the development of successful remedies for sediment sites. The present article highlights concepts and considerations that are necessary for deriving representative background concentrations (including both anthropogenic and natural concentrations) to achieve a more complete understanding of historical and ongoing sources to the site. In the absence of detailed guidance, these considerations may be overlooked or discounted when calculating representative background concentrations at sediment sites.

Conceptual site models are critical tools for characterizing the complexity of sources to a site, migration pathways, receptors, and exposure pathways, and they inform the appropriate selection of background reference areas. The CSM developed for a site should include these key considerations (as outlined in the present article):

- Anthropogenic inputs to the site, such as land use, urban runoff, direct discharge, sediment transport, atmospheric deposition, and source control.
- Natural characteristics of a site, such as sediment physical properties, hydrodynamic environment, sediment profile, and geochemistry.

Similarities between the site and the background reference areas are important because they influence the transport and fate of contamination. Anthropogenic sources that cannot be controlled contribute ongoing contamination and should be fully considered and incorporated into the CSM because they represent anthropogenic background chemical concentrations that will persist on site during and after any remedy. It may not be feasible to control all offsite sources of anthropogenic background prior to remediation, which should inform potential cleanup goals.

After selecting representative background reference areas and completing a targeted sampling program that uses sampling methods matching those used during site data collection, the data should be closely evaluated. The focus of the data evaluation should be on comparing site data with background data, using appropriate statistical approaches (along with geochemical evaluation for trace metals) to derive technically defensible representative background concentrations. During the data evaluation, it is imperative that false outliers are retained and are not arbitrarily removed because natural variability occurs in a data set. A statistically appropriate outlier evaluation should be performed on the background data set, and the evaluation should include the key steps outlined in the discussion on outlier evaluation. Of critical importance, only true outliers should be removed from data sets, and false outliers should not be arbitrarily eliminated.

Representative background concentrations should remain fixed for the duration of the remedial investigation and remedial response. Otherwise, the lack of certainty for stakeholders would be an impediment to the implementation of any remedy. Collectively, the considerations and approaches outlined in the present article should increase the ability to derive technically defensible representative background concentrations. These recommendations are offered to help inform, improve, and increase the consistency of sediment site remedy decision making.

## Disclaimer

The peer‐review process for this article was managed by the Editorial Board without the involvement of S Brown.

## SUPPLEMENTAL DATA

Adapted from Important Considerations in the Derivation of Representative Background Concentrations for the Evaluation of Sediment Sites: Supplemental Material Related to a Conceptual Site Model

## Supporting information

This article includes online‐only Supplemental Data.

Supporting Information

## Data Availability

No analytical data were used in the preparation of this article.
