# Supplementary material for: Important considerations in the derivation of background at sediment sites
Source: Integr Environ Assess Manag. 2019 Mar 4;15(3):448–57. doi: 10.1002/ieam.4124 (PMC6850622; doi:10.1002/ieam.4124)
Supplement: Supplementary file 1 — Supporting Information [file IEAM-15-448-s001.docx]

**Adapted from Important Considerations in the Derivation of Representative Background Concentrations for the Evaluation of Sediment Sites:
Supplemental Material Related to a Conceptual Site Model**

Allison Geiselbrecht, Shahrokh Rouhani, Karen Thorbjornsen, Douglas Blue, Steven Nadeau,
Tessa Gardner-Brown, Steven Brown

May 2018

*This document provides supplemental material to the associated journal article, with focus on elements of the conceptual site model and its importance in the derivation of representative background concentrations for the evaluation of sediment sites.*

# 1.0 Elements of a Conceptual Site Model

Background reference areas selected for derivation of representative background concentrations should be as similar to the site as possible, except for site-related releases. Therefore, developing a robust CSM will help to ensure that the selected background reference areas are similar to the site and will inherently provide an increased understanding of the factors that may contribute to representative background concentrations at the site. Factors that typically contribute to representative background concentrations (and chemical concentrations on a site, as would likely be shown through the CSM) are detailed in this Supplemental Material document. These factors should be considered when developing the study design for representative background determination.

Figure 1 presents a simplified CSM for a sediment site, focusing on the anthropogenic inputs and natural characteristics outlined in this section. Importantly, Figure 1 does not depict complex interactions, such as the cycling of chemicals of concern within the environmental system, which can be important at some sites. Additionally, this supplemental material is not inclusive of all possible chemical fate/transport and exposure pathways that may be relevant to the derivation of representative background concentrations at different sites (e.g., groundwater-surface water interactions, spills).


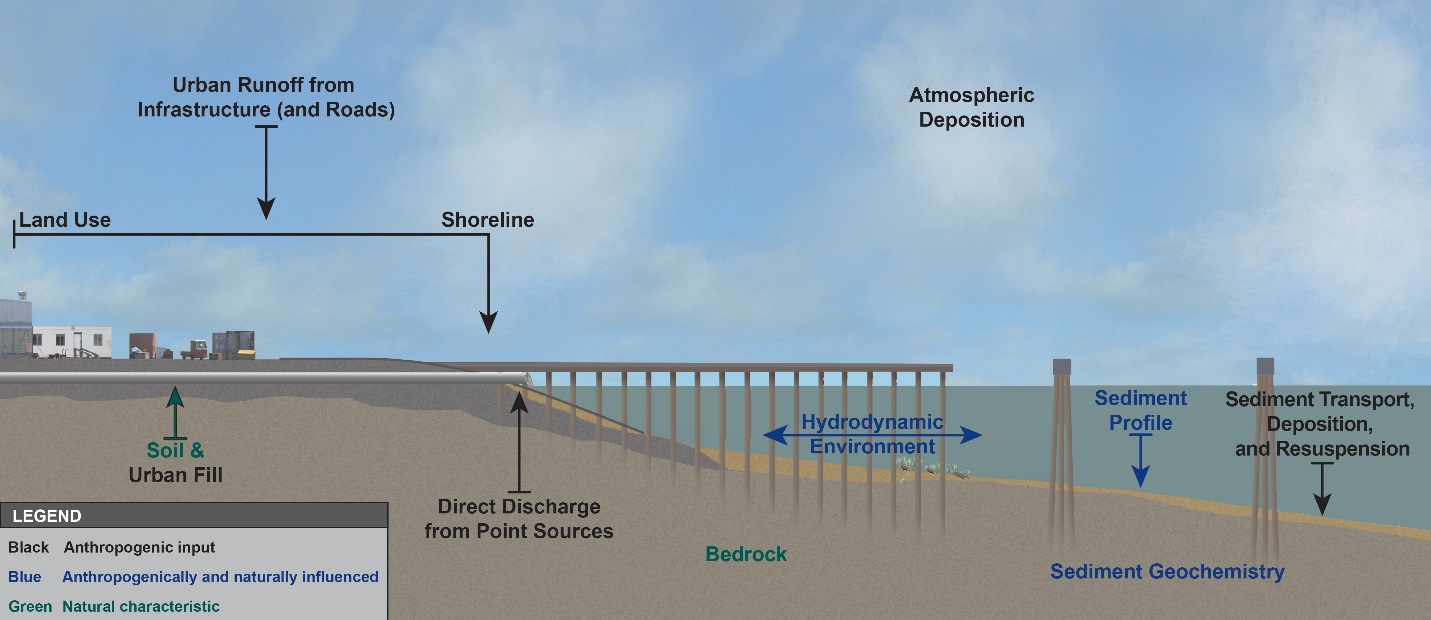
Figure 1. Key Considerations in Development of a Simplified Conceptual Site Model

Sections 1.1 through 1.6 discuss some of the key complexities that are encountered during the CSM development process at sediment sites, and the discussion has been developed for use by a broad audience. Some of the more specific technical considerations, such as evaluation of total organic content and grain size and their specific relationship to organic compounds and data treatment, are not discussed specifically within the following sections. However, the overarching principle is that a robust CSM assists in selection of representative background areas, and these background areas need to reflect the site as closely as possible, with the exception of site-related inputs.

## 1.1 Land Use within a Watershed

Several studies have demonstrated that the degree of urbanization, intensity of land use, and land cover patterns adjacent to a site (or background reference areas) are correlated with chemical concentrations. Generally, a practitioner should recognize that contaminant concentrations tend to increase as the degree of urbanization increases (Nowell *et al.* 2013). The degree of urbanization positively correlates to the level of chemical input that can be expected to migrate onto the site before, during, and after the completion of the remedy; so this should be considered in selecting background reference areas and determining representative background concentrations.

### 1.1.1 Degree of Urbanization

Contamination associated with urbanization moves through the environment via a variety of transport pathways, including surface water transport, urban runoff, bank erosion, and sediment resuspension, among others.

The USGS has evaluated chemical concentrations in watersheds across different degrees of urbanization, in order to better understand the correlation between urban land use and chemical concentrations. These studies have shown that environmental media in more urbanized areas contain elevated concentrations of chemicals compared to less urban areas (Nowell *et al*. 2013, Kemble *et al*. 2013). These studies concluded that concentrations of a wide range of contaminants, including polycyclic aromatic hydrocarbons (PAHs), PCBs, organochlorine pesticides, and metals, were “significantly related to urbanization across the study areas” (Nowell *et al*. 2013).

Consequently, historical and current land use within a watershed has a direct and potentially major influence on anthropogenic background conditions (i.e., chemical concentrations). The type and intensity of land use surrounding selected background reference areas should be as similar as possible to that observed at the site, to account for chemical input that is associated with urbanization. This practice will ensure that anthropogenic background concentrations reflect the level of contamination that is generally associated with land use in the vicinity of the site, absent contributions from releases from the site itself. This practice will also help facilitate the derivation of representative background concentrations for determining achievable cleanup goals.

### 1.1.2 Shoreline Conditions

Shoreline condition should be evaluated as part of CSM development (USEPA 2005) when screening and selecting background reference areas. Waterfront development, particularly for industrial purposes, typically includes hardened shorelines such as sheetpile walls, bulkheads, or riprap slopes. Hardened shorelines protect against erosion, but may be susceptible to sediment contaminant migration through sheetpile seams and holes in older steel, due to corrosion or accidental puncture. Unprotected shorelines are more susceptible to erosion from upland runoff, tidal action, and storm surge, releasing soils that may be impacted by site-related activities to the water body.

A number of examples of recontamination at Superfund sites due to contaminated soil erosion (e.g., slumping under docks and scouring after high flow storm events) are described by the Association of State and Territorial Solid Waste Management Officials in *Sediment Remedy Effectiveness and Recontamination: Selected Case Studies* (ASTSWMO 2013). Case studies described include the Torch Lake/Quincy Smelter Site in Michigan and the Denny Way Combined Sewer Overflow (CSO) Site in Washington, where continuing shoreline erosion has negatively impacted the remedies.

Alternatively, natural shorelines may indicate lower levels of land use intensity and could result in a source of less impacted eroding material entering the water column. Potential migration of impacted bank soil into adjacent sediments should be considered, because this migration could impact chemical concentrations in adjacent sediments and downstream.

Finally, floodplains and marshes within sites (or near the site), especially when tidally influenced, are particularly challenging. These features can cover a large surface area, usually have complex patterns of erosion and deposition, and the location of the shoreline is constantly changing. As such, inputs from these areas are often critical components in developing a robust CSM for the site.

## 1.2 Watershed Inputs

Sediment sites are predominantly affected by historical chemical contributions and point-source releases. These sites are often located within urban areas, with multiple potential sources of additional and on-going chemical inputs from point and non-point sources that are unrelated to the site. Sources of contamination that are not site-related, but are from within the watershed, both historical and current, may include many of the same chemicals being studied at the subject sediment site, making it difficult to discern between site-related releases and inputs from background sources.

For example, almost half of the largest sediment sites have PCBs as a major contaminant, and approximately a quarter or more are contaminated with metals and/or PAHs from legacy or point-source releases (USEPA 2005). These contaminants are also ubiquitous in the urban environment and are transported through urban runoff, atmospheric deposition, and direct discharges from outfalls (municipal and/or industrial). Consequently, it is critical to recognize that these ongoing sources will continue to contribute contaminant concentrations to background reference areas and the site. Thus, these sources should be included in determining representative background concentrations for these background reference areas.

### 1.2.1 Urban Runoff

Urban runoff is non‐point source pollution defined as “stormwater from city streets and adjacent domestic or commercial properties that carries pollutants of various kinds into the sewer systems and receiving waters” (USEPA 2010). Urban runoff is considered to be a significant contributor of contamination to watersheds and sediments, and contains many chemicals most commonly found at sediment sites (PCBs, PAHs, and metals), as noted in Section 1.2 (USEPA 1995).

Urban runoff also contains chemicals that are commonly found in urban infrastructure, including asphalt roads, pavement sealants, building materials, roofing materials, and galvanized fences. For example, a recent study by the USGS and the Milwaukee Metropolitan Sewage District stated that “coal-tar pavement sealant was indicated as the primary source of PAHs in a majority of streambed sediment samples, contributing an estimated 77 percent of total PAHs to samples, on average” (Baldwin *et al*. 2016). Releases to the environment are also attributable to motor vehicle use; wear on automotive parts (e.g., tires, brake pads), and vehicle emissions (Chalmers *et al*. 2007, Gallagher *et al*. 2014, Turner and Hallett 2012). Other sources of different chemicals, such as home pesticide application and improper waste disposal, also contribute to chemical concentrations in urban runoff.

Therefore, contributions and chemical loading from urban runoff to a site should also be included in the development of representative background concentrations. Chemical inputs from urban runoff to a site and background reference areas should be as similar as possible in order to obtain representative background concentrations for use at the site.

### 1.2.2 Direct Discharges

In general, direct discharges are associated with industrial facilities, or municipally owned systems that discharge wastewater and/or stormwater to water bodies through permitted (or unpermitted) conveyance systems, via discharge points such as outfalls and CSOs. Chemical loading from wastewater and/or stormwater discharge is managed by the National Pollutant Discharge Elimination System (NPDES) program under the Clean Water Act, which may set limits for chemical concentrations for discharges from these conveyance systems, but does not completely eliminate chemical loading from the discharge. Additionally, the regulatory programs may not measure or exercise authority over chemicals associated with sediment sites, such as PCBs.

In 2006, a Phthalates Work Group (Work Group) was formed by the USEPA and state and local agencies to address recontamination from phthalates observed in the Thea Foss Waterway located within the Commencement Bay Superfund Site in Tacoma, Washington. A key conclusion identified by the Work Group is that “rapid accumulation of phthalates in sediments (after cleanup) is associated with urban stormwater outfalls” (Floyd|Snider 2007). In particular, the head of the Thea Foss Waterway has two 96-inch-diameter stormwater pipes that continuously discharge untreated and treated industrial stormwater, in addition to untreated residential stormwater. These outfalls were determined to be a main source of phthalate input, and the resulting recontamination to the Thea Foss Waterway, particularly in the vicinity of the outfalls (ASTSWMO 2013).

Chemical loading to a site from direct discharges should be accounted for in representative background concentrations. “In some cases, as part of a response to address CERCLA releases of hazardous substances, pollutants, and contaminants, USEPA may also address some of the background contamination that is present on a site due to area-wide contamination” (USEPA 2002). As much as practical, direct discharges affecting background reference areas should be as closely matched as possible to the direct discharges affecting a site. Municipalities overseeing wastewater treatment plant and CSO discharges within a waterbody undergoing sediment cleanup may have data on chemical concentrations in the treatment plant and CSO discharges, which can also be useful in the derivation of representative background concentrations.

### 1.2.3 Sediment Transport

Sediment sites are dynamic in nature, as they are consistently receiving suspended sediments from off-site areas. Those off-site areas contain background concentrations of contaminants from anthropogenic sources, and may also contain concentrations of naturally occurring chemicals similar to the chemicals of concern for the site. At the Lower Duwamish Waterway Superfund site, sediment transport modeling performed as part of the Remedial Investigation/Feasibility Study indicated that “approximately 99 percent of the total external sediment particle load to the Lower Duwamish Waterway comes from the Green River, upstream of the Lower Duwamish Waterway” (Windward 2010).

An analysis of suspended sediments collected upstream of the Lower Duwamish Waterway site performed by the Washington State Department of Ecology indicated that this loading could potentially be a post-remedy source of recontamination to sediments (Ecology 2009), because the upstream sediment contains chemicals (such as PCBs) that are found at high concentrations throughout the downstream site. A dredge and backfill early action remedy was conducted along the Lower Duwamish Waterway between 2013 and 2015. Within months of completing the remedy, high levels of PCBs were measured in material deposited on the clean sediment surface, with concentrations much greater than what was predicted to occur. Similar post-remedial recontamination was also observed at locations where other early actions were performed on the Duwamish (AMEC Foster Wheeler 2017).

Background reference areas in non-tidal riverine systems are frequently located immediately upstream of the site. In tidally influenced sites, the situation is considerably more complex. At tidally influenced sites, sediment transport into the site may result from upstream sources and may also involve contributions from receiving bodies downstream of the site as sediments are transported on incoming tides. Therefore, within tidal systems it is extremely important to have a strong understanding of hydrodynamics and sediment transport processes. If downstream sediments contain equal or greater concentrations of contaminants than are found at the site, these downstream sediments can be a continuing source of contaminant input to the site and should be considered in developing representative background concentrations.

Because sediment resuspension is a transport pathway for contamination, it is important to acknowledge that representative background levels of contamination will inevitably move into a site through this natural process. It is also important to understand the diversity of depositional environments and the many varying factors such as current directions, tidal pumping, and constant or episodic sediment transport processes. Consideration should be given to multiple potentially significant factors influencing sediment transport at coastal/tidal sites, including the effects of flood events and storm surges. Among these, downstream flows intersect with the tide to create a salt wedge and an estuarine turbidity maximum where dissolved materials flocculate and deposit. This effect, combined with suspended particulate material, creates a locally elevated area of turbidity that moves through the estuary and contributes to sediment transport and deposition. Finally, in some riverine or estuarine systems, the current can reverse direction and head upriver, under certain circumstances.

### 1.2.4 Atmospheric Deposition

Atmospheric deposition from industrial and urban areas, and areas near major transportation corridors, is a recognized pathway of contamination, particularly for those contaminants ubiquitously found in the environment; these include metals, PAHs, PCBs, and pesticides, as discussed in Section 1.2 (Larson *et al*. 1997, ESA 2000, Landis and Keeler, 2002, Rolfhus *et al*. 2003, Kuang *et al*. 2003, USGS 2005, Urbaniak 2007, Brandenberger *et al*. 2010, Zhang *et al*. 2013, Amodio *et al*. 2014). The impact of atmospheric deposition can be challenging to ascertain. Types and volumes of pollutants deposited from the atmosphere will vary depending on atmospheric conditions (e.g., wind speed, temperature, and rainfall) and particle characteristics (size and shape). The influence of these factors on the resulting contaminant deposition rate may vary (Amodio *et al*. 2014).

Winds can carry chemicals through the air from great distances, further confounding the identification and control of non-point sources of contamination (Cohen *et al*. 1997). At sites with relatively uniform sources of atmospheric deposition (e.g., transportation corridors and urbanized, non-industrial areas), typical concentrations and mass loading effects are usually established through a literature review. Additionally, sites in industrial areas with contributions of airborne chemicals may warrant further consideration of site-specific variations (e.g., physical and chemical characteristics of specific industrial emissions, localized wind patterns) that influence deposition patterns, which may not be readily apparent during a literature review.

In Washington State, studies of air deposition and resulting mass flux loading have generally concluded that air deposition is a small but potentially significant source of certain persistent chemicals, and may account for up to 5 percent of the measured concentration of any particular chemical in sediment that is well outside of the influence of urbanized areas (Brandenberger *et al.* 2010). Similar studies of atmospheric deposition in the Great Lakes region have concluded that atmospheric deposition is a significant source of mercury and some other trace metals to Lake Michigan and Lake Superior (Landis and Keeler 2002, Rolfhus *et al.* 2003). Studies of zinc loading to the Santa Monica Bay determined that atmospheric deposition was responsible for 62 percent of the measured zinc concentration in sediments (ESA 2000). The Delaware River Basin Commission found that “air concentrations of PCBs in the region currently are two orders of magnitude above the concentration required to achieve equilibrium and halt contributions of PCBs from the air to the water” (Fikslin and Suk 2003). Substantial additional literature is available documenting the contributions of air deposition to elevated chemical concentrations in surface sediment. The contribution of chemicals from this pathway should be recognized both at a site, and at its background reference areas; in fact, background reference areas should reflect atmospheric deposition conditions observed at the site.

## 1.3 Source Control

Source control is generally defined as efforts to eliminate or reduce, to the extent practicable, the release of chemicals from point and non-point sources to a water body (USEPA 2005). Source control measures vary, depending on the transport pathway. For example, reducing contamination from urban runoff typically requires different measures than those used to reduce contamination from direct discharges, although the efforts may be coordinated.

Source control should be fully complete, or at least substantially completed, before remediation of a sediment site begins. If source control has not been completed or is not feasible, then it is critical that the potential inputs from uncontrolled ongoing sources be included in the determination of representative background concentrations, because these inputs would continue to affect the site after remediation and that recontamination of the completed remedy would occur. For example, at a riverine site there may be substantial ongoing CSO contributions upstream of the site boundary, or from within the site itself. If the municipality responsible for the CSOs is not able to implement source control prior to remediation of the sediment site, the CSO input must be included and represented in the derivation of representative background concentrations, as the input from these point sources will continue into the future, after completion of the remedy.

In general, it is important to recognize that, while source control is key, in many sites it may be impossible to eliminate source contributions altogether. This is particularly the case at urbanized and/or tidally influenced sites. The inability to eliminate ongoing source contributions makes it all the more critical to take ongoing sources into account when setting representative background concentrations for the site.

## 1.4 Sediment Physical Properties

The physical properties of sediment strongly influence the distribution of naturally occurring and anthropogenic background chemicals in the environment. Sediment consists of organic material, inorganic material, and pore water. The relative abundance of these components varies vertically and horizontally within a sediment body, resulting in variable distribution of chemicals at a sediment site. Metals concentrations, in particular, can be heavily influenced by natural processes. Since representative background includes natural sources (as well as anthropogenic sources), a discussion related to contributions of natural background is included within this Supplemental Material.

The organic fraction has an important effect on the concentration of chemicals, because of its high capacity for sorption of some contaminants. The water fraction fills pore space within the sediment, allows for the transport of dissolved chemicals, and is subject to geochemical conditions that strongly influence the transport and sorption of metals (refer to Section 1.6). The inorganic fraction typically makes up the largest portion of sediment mass; the relative fractions of sand, silt, and clay determine the sediment texture.

To accurately quantify sediment characteristics, geotechnical testing and general chemistry analyses are generally recommended, these should be conducted according to ASTM International geotechnical testing standards and USEPA analytical methods. The sediment type (ASTM 2009), particle size (ASTM 2017a, 2017b), density (ASTM 2017c), and moisture content (ASTM 2010) should be the focus of geotechnical tests. The general chemistry analyses that are recommended include sediment pH (USEPA 2000), oxidation-reduction conditions (redox potential; APHA 2011), and total organic carbon (TOC) content (USEPA 1999).

Sediment texture has a substantial effect on the distribution of chemicals of concern in sediment. Several grain size classifications are available for soil classification, and the Unified Soil Classification System (USCS; ASTM 2011) is most commonly used to classify sediments. Fine-grained sediments, particularly those with a high percentage of clay-sized particles and organic content (as measured by TOC) have greater surface area, so they typically have greater sorption capacity for some contaminants than medium and coarse-grained sediments. Silts typically have moderate sorption capacity, while sands and gravels have lower sorption capacity.

In addition to sediment texture, sorption capacity of some fine-grained sediment is enhanced by surface charge. For example, clays and organic colloids tend to be highly charged relative to their surface areas. Clay minerals are typically negatively charged under normal pH conditions, so they attract positively charged trace metals ions for sorption. This results in clay-rich, fine-grained sediments that have greater trace and reference metal concentrations (refer to Section 1.6). In addition, metal concentrations (in particular) tend to be inversely proportional to grain size.

Given the ability of sediment physical properties to influence the distribution of chemicals in the environment, this is an important consideration in developing a CSM to support representative background determination.

## 1.5 Hydrodynamic Environment and Sediment Profile

Sediment characteristics that strongly influence the distribution of both naturally occurring and anthropogenic background chemicals are determined chiefly by the physical configuration and hydrodynamic characteristics of the depositional environment. In general, coarse-grained sediments such as sands are deposited in relatively high-energy environments (such as beaches and river channels), while fine sediments settle out only when they reach lower energy areas (such as offshore, lakes, and more quiescent areas of rivers and streams). As described in Section 1.4, fine sediments typically have a greater sorption capacity for contaminants than coarse-grained sediments, so representative background concentrations tend to be greater in more quiescent sediment environments, where the percentage of fine sediments is greater.

The vertical profile of sediment may vary significantly in composition, texture, chemical, and biological characteristics. Changes in the hydrodynamic environment and sediment sources can result in distinct layering. Change in land use over time, such as increasing urbanization, may produce layers with different compositions, texture, and concentrations of anthropogenic background chemicals. Natural or artificial changes to vegetation within a watershed may alter the concentration of organic carbon in sediment layers. All of these factors can influence the distribution of chemicals at a site and within its background reference areas.

The rates of sediment deposition, erosion and removal, and mixing vary widely among aquatic environments and should also be assessed as part of the CSM, as these factors affect chemical distribution in sediments. Pore space and volume of the water within sediments is decreased by compaction as sediments are buried. During this process, chemicals present in sediments may be vertically redistributed by mixing of surface and deeper sediments. Sediment mixing may also occur through bioturbation. Bioturbation may increase pore space, the volume of the water fraction, and organic content, and affect the partitioning of chemicals between aqueous and solid phases.

Age dating and chemical analysis of sediment core samples may indicate chemical concentrations that are associated with sediment layers deposited prior to site-related activities, which may be helpful in estimating representative background concentration ranges. The age of sediment layers and accumulation rates may be estimated by various methods, including radioisotopic decay measurements (USGS 1998).

## 1.6 Geochemistry

It is important to identify the geochemical processes controlling element concentrations in sediment samples. Sediment geochemistry should be characterized by properly qualified geochemists in support of background analysis (e.g., to determine which samples to retain in the background dataset) and should be considered during subsequent comparisons of site versus background datasets. This is also important because metal concentrations (either naturally occurring, or from an anthropogenic source other than a release at a site) commonly exceed risk-based screening criteria. Geochemical processes relevant to background data evaluation include association of elements with minerals, sorption of elements on mineral surfaces, water chemistry, and water-mineral interactions. These topics are summarized in this section. Geochemical methods used for evaluating representative background metal concentrations in sediment are discussed further in the associated journal article.

Chemical properties of sediment particles and the surrounding aqueous phase strongly influence the distribution of metals, and for this reason are useful to measure and include in geochemical assessment of site data. Key properties include metal solubility, pH of the aqueous phase (including the overlying water column and pore water), redox potential of the aqueous phase, metal affinity for organic carbon, TOC concentrations, and reactions of metals with sulfide.

Metals concentrations are controlled by dissolution/precipitation reactions and adsorption/desorption (“sorption”) reactions. Highly soluble metals can remain mobile in the aqueous phase and can be dissolved from the sediment, while low solubility metals can precipitate and accumulate in sediments. The solubility of a metal is highly dependent on characteristics of the aqueous phase including pH, redox potential, and ionic strength. While elements differ in their response to changing pH, acidic conditions tend to dissolve and mobilize some metals, while basic pH conditions can precipitate other metals (resulting in greater concentrations in sediment). The pH also controls the net surface charge of particles, which is an important factor in determining sorption of metals on mineral surfaces. This is important due to the presence of clay minerals and metal oxides that have strong affinities to absorb specific trace metals.

Oxidizing conditions cause many metal ions (e.g., iron and manganese) to precipitate as oxides. Reducing conditions, such as in anoxic sediments, tend to keep specific elements in solution and mobile. In addition to naturally reducing conditions associated with peat or other organic materials in wetlands or similar environments, releases of organic contaminants can stimulate microbial activity, resulting in local reducing conditions and the mobilization of select metals.

Reducing conditions can cause the reductive dissolution of iron and manganese oxides, which may mobilize adsorbed trace elements. Reducing conditions may also directly reduce arsenic, selenium, antimony, molybdenum, and vanadium to more mobile valence states. Sulfate-reducing conditions in sediment can cause specific metals (e.g., arsenic, mercury, copper, lead, and zinc) to precipitate as (or with) insoluble sulfide phases. Sulfide ions, produced from the reduction of sulfate associated with the breakdown of organic compounds and as measured by acid-volatile sulfide, are usually associated with higher metals concentrations in sediment.

As noted previously, sediments with greater TOC concentrations typically have greater concentrations of specific trace elements (e.g., mercury, copper, tin, and uranium), because the organic particles have a greater sorptive capacity for these elements. However, greater TOC concentrations may be associated with reducing conditions, so the metals associated with the TOC may be less bioavailable.

The complexity of contaminant interactions, as overviewed in this section, may hinder the ability to identify the background reference areas with the same sediment geochemistry. For that reason, and the other reasons discussed throughout this document, it may be appropriate to identify and utilize multiple background reference areas in order to define a range of reference conditions.

# 2.0 References

Amodio, M., S. Catino, P.R. Dambruoso, G. de Gennaro, A. Di Gilio, P. Giungato, E. Laiola, A. Marzocca, A. Mazzone, A. Sardaro, and M. Tutino. 2014. “Atmospheric Deposition: Sampling Procedures, Analytical Methods, and Main Recent Findings from the Scientific Literature.” *Advances in Meteorology*, Vol. 2014, Article ID 161730. <<http://dx.doi.org/10.1155/2014/161730>>. 22 June.

AMEC Foster Wheeler. 2017. *Additional Duwamish Sediment Other Area Backfill Sampling Data Report: Duwamish Sediment Other Area and Southwest Bank Corrective Measure and Habitat Project, Boeing Plant 2, Seattle/Tukwila, Washington.* Prepared for the Boeing Company. July. (Pending; currently under USEPA-review)

American Public Health Association (APHA). 2011. *APHA Method 2580 Oxidation-Reduction Potential (ORP): Standard Methods for the Examination of Water and Wastewater*. 40 CFR 141.121. Prepared and published jointly by the American Public Health Association, American Water Works Association, and Water Environment Federation. Editorial revisions 2011.

Association of State and Territorial Solid Waste Management Officials (ASTSWMO). 2013. *Sediment Remedy Effectiveness and Recontamination: Selected Case Studies*. Prepared by the ASTSWMO Sediments Focus Group with assistance from the U.S. Environmental Protection Agency. <<https://clu-in.org/download/contaminantfocus/sediments/2013-04-Sediment_Remedy_Effectiveness_and_Recontamination.pdf>>. April.

ASTM International (ASTM). 2009. *ASTM D2488-09a, Standard Practice for Description and Identification of Soils (Visual-Manual Procedure)*. ASTM International, West Conshohocken. <<https://www.astm.org/Standards/D2488.htm>>.

_____. 2010. *ASTM D2216-10, Standard Test Methods for Laboratory Determination of Water (Moisture) Content of Soil and Rock by Mass*. ASTM International, West Conshohocken, PA. <<https://www.astm.org/Standards/D2216.htm>>.

_____. 2011. *ASTM D2487-11, Standard Practice for Classification of Soils for Engineering Purposes (Unified Soil Classification System)*. ASTM International, West Conshohocken, PA. <<https://www.astm.org/Standards/D2487.htm>>.

_____. 2017a. *ASTM D6913 / D6913M-17, Standard Test Methods for Particle-Size Distribution (Gradation) of Soils Using Sieve Analysis*. ASTM International, West Conshohocken, PA. <<https://www.astm.org/Standards/D6913.htm>>.

_____. 2017b. *ASTM D7928-17, Standard Test Method for Particle-Size Distribution (Gradation) of Fine-Grained Soils Using the Sedimentation (Hydrometer) Analysis.* ASTM International, West Conshohocken, PA. <<https://www.astm.org/Standards/D7928.htm>>.

_____. 2017c. *ASTM D2937-17e1, Standard Test Method for Density of Soil in Place by the Drive-Cylinder Method*. ASTM International, West Conshohocken, PA. <<https://www.astm.org/Standards/D2937.htm>>.

Baldwin, Austin K., Steven R. Corsi, Michelle A. Lutz, Christopher G. Ingersoll, Rebecca A. Dorman, Christopher Magruder, and Matthew Magruder 2016. “Primary sources and toxicity of PAHs in Milwaukee-area streambed sediment.” *Environmental Toxicology and Chemistry.* <<http://onlinelibrary.wiley.com/doi/10.1002/etc.3694/full>>. 22 December.

Brandenberger, J.M., P. Louchouarn, L-J Kuo, E.A. Crecelius, V. Cullinan, G.A. Fill, C. Garland, J. Williamson, and R. Dhammapala. 2010. *Control of Toxic Chemicals in Puget Sound, Phase 3: Study of Atmospheric Deposition of Air Toxics to the Surface of Puget Sound.* Prepared for Washington State Department of Ecology Air Quality Program. Publication No. 10-02-012. <<https://fortress.wa.gov/ecy/publications/documents/1002012.pdf>>. July.

Chalmers, A.T., P.C. Van Metre, and E. Callender. 2007. “The chemical response of particle associated contaminants in aquatic sediment to urbanization in New England, U.S.A.” *Journal of Contaminant Hydrology* 90(1-2): 4–25.

Cohen, Mark, Paul Cooney, and Barry Commoner. 1997. *The Transport and Deposition of Persistent Toxic Substances to the Great Lakes: V. Summary.* Prepared for the International Joint Commission’s International Air Quality Advisory Board. <<http://www.arl.noaa.gov/documents/reports/cohen/05_Summary.pdf>>. December.

Ecological Society of America (ESA). 2000. *Where Air and Water Meet Atmospheric Deposition to the Pacific Coast: Workshop Report 2000*. <[https://www.esa.org/esa/
science/reports/atmospheric-deposition/](https://www.esa.org/esa/science/reports/atmospheric-deposition/)>.

Ecology, Washington State Department of (Ecology). 2009. *Contaminant Loading to the Lower Duwamish Waterway from Suspended Sediment in the Green River*. Publication No. 09-03-028. Prepared by the Toxics Studies Unit, Environmental Assessment Program. <<https://fortress.wa.gov/ecy/publications/documents/0903028.pdf>>. November.

Fikslin, T.J., and N.S. Suk. 2003. *Total Maximum Daily Loads for Polychlorinated Biphenyls (PCBs) for Zones 2 - 5 of the Tidal Delaware River.* Delaware River Basin Commission, West Trenton, NJ. <<http://www.nj.gov/drbc/library/documents/TMDL/FinalRptDec2003.pdf>>. December.

Floyd|Snider. 2007. Sediment Phthalates Work Group Meeting Notes November 29, 2006. <<http://52.33.28.53:8004/programs/tcp/smu/phthalates/Occurrence.pdf>>. 31 January.

Gallagher, Matthew T., Joel W. Snodgrass, Adrianne B. Brand, Ryan E. Casey, Steven M. Lev, and Robin J. Van Meter. 2014. “The role of pollutant accumulation in determining the use of stormwater ponds by amphibians.” *Wetlands Ecology and Management* 22(5): 551−564.

Kemble, Nile E., Douglas K. Hardesty, Christopher G. Ingersoll, James L. Kunz, Paul K. Sibley, Daniel L. Calhoun, Robert J. Gilliom, Kathryn M. Kuivila, Lisa H. Nowell, and Patrick W. Moran. 2013. “Contaminants in Stream Sediments From Seven United States Metropolitan Areas: Part II—Sediment Toxicity to the Amphipod *Hyalella azteca* and the Midge *Chironomus dilutus*.*”* *Environmental Contamination and Toxicology* 64 (1): 52–64.

Kuang, Z., L.L. McConnell, A. Torrents, D. Meritt, and S. Tobash. 2003. “Atmospheric deposition of pesticides to an agricultural watershed of the Chesapeake Bay.” *Journal of Environmental Quality* 32(5):1611–1622. <[https://pubag.nal.usda.gov/pubag/
downloadPDF.xhtml?id=27260&content=PDF](https://pubag.nal.usda.gov/pubag/downloadPDF.xhtml?id=27260&content=PDF)>.

Landis, Mathew S., and Gerold J. Keeler. 2002. “Atmospheric mercury deposition to Lake Michigan during the Lake Michigan Mass Balance Study.” *Environmental Science and Technology* 36(21): 4518–4524.

Larson, Steven J., Paul D. Capel, and Michael S. Majewski. 1997. *Pesticides in Surface Water: Distributions, Trends, and Governing Factors*. Chelsea, Michigan: Ann Arbor Press Inc. 1 November.

Nowell, Lisa H., Patrick W. Moran, Robert J. Gilliom, Daniel L. Calhoun, Christopher G. Ingersoll, Nile E. Kemble, Kathryn M. Kuivila, and Patrick J. Phillips. 2013. “Contaminants in Stream Sediments From Seven United States Metropolitan Areas: Part I: Distribution in Relation to Urbanization.” *Archives of Environmental Contamination and Toxicology*. 64: 31–51. 6 November.

Rolfhus, K.R, H.E. Sakamoto, L. B. Cleckner, R.W. Stoor. C.L. Babiarz, R.D. Back, H. Manolopoulos, and J.P. Hurley. 2003. “Distribution and Fluxes of Total and Methylmercury in Lake Superior.” *Environmental Science and Technology* 37(5): 865–872.

Turner, Andrew, and Luke Hallett. 2012. “Bioaccessibility of Zinc in Estuarine Sediment Contaminated by Tire Wear Particles.” *Water, Air, & Soil Pollution* 223(8): 4889−4894.

Urbaniak, Magdalena. 2007. “Polychlorinated Biphenyls: Sources, Distribution and Transformation in the Environment—A Literature Review.” *Acta Toxicologica* 15(2):
83–93.

U.S. Environmental Protection Agency (USEPA). 1995. *National Water Quality Inventory: 1994 Report to Congress*. Publication No. EPA 841-A-95-001. <[https://www.epa.gov/sites/production/files/2015-09/documents/1994_
national_water_quality_inventory_report_to_congress.pdf](https://www.epa.gov/sites/production/files/2015-09/documents/1994_national_water_quality_inventory_report_to_congress.pdf)>. December.

_____. 1999. *Total Organic Carbon (TOC) in Soil: SW-846 Method 9060*. <[https://www.epa.gov/
sites/production/files/2015-06/documents/9060dqi.pdf](https://www.epa.gov/sites/production/files/2015-06/documents/9060dqi.pdf)>. 16 November (revised).

_____. 2000. *pH in Liquid and Soil: SW-846 Method 9040 (Liquid) and SW-846 Method 9045 (Soil)*. <<https://www.epa.gov/sites/production/files/2015-06/documents/9045_1crf.pdf>>. 21 January (revised).

_____. 2002. *Guidance for Comparing Background and Chemical Concentrations in Soil for CERCLA Sites*. Prepared by the Office of Emergency and Remedial Response. Publication No. EPA 40-R-01-003 and OSWER 9285.7-41. <[http://dec.alaska.gov/spar/csp/
guidance_forms/docs/background.pdf](http://dec.alaska.gov/spar/csp/guidance_forms/docs/background.pdf)>. September.

_____. 2005. *Contaminated Sediment Remediation Guidance for Hazardous Waste*.
Prepared by the Office of Emergency and Remedial Response. Publication
No. EPA-540-R-05-012 and OSWER 9355.0-85. <[https://clu-in.org/download/
contaminantfocus/sediments/contaminated-sediment-remediation-EPA-guidance.pdf](https://clu-in.org/download/contaminantfocus/sediments/contaminated-sediment-remediation-EPA-guidance.pdf)>. December.

_____. 2010. Vocabulary Catalog: Aquatic Biodiversity Glossary. <[https://ofmpub.epa.gov/
sor_internet/registry/termreg/searchandretrieve/glossariesandkeywordlists/search.do?details&glossaryName=Aquatic+Biodiversity+Glossary](https://ofmpub.epa.gov/sor_internet/registry/termreg/searchandretrieve/glossariesandkeywordlists/search.do?details&glossaryName=Aquatic+Biodiversity+Glossary)>. 8 December.

U.S. Geological Survey (USGS). 1998. *Short-Lived Isotopic Chronometers, A Means of Measuring Decadal Sedimentary Dynamics*. Fact Sheet FS-73-98. <[https://pubs.usgs.gov/
fs/1998/0073/report.pdf](https://pubs.usgs.gov/fs/1998/0073/report.pdf)>.

_____. 2005. *Contribution of Atmospheric Deposition to Pesticide Loads in Surface Water Runoff.* <<https://pubs.usgs.gov/of/2005/1307/ofr2005_1307.pdf>>.

Windward Environmental, LLC (Windward). 2010. *Lower Duwamish Waterway Remedial Investigation Report, Final.* Prepared on behalf of the Lower Duwamish Waterway Group. <<http://ldwg.org/Assets/Phase2_RI/Final%20RI/Final_LDW_RI.pdf>. 9 July.

Zhang, Xianming, Torsten Meyer, Derek C.G. Muir, Camilla Teixeira, Xiaowa Wang, and Frank Wania. 2013. “Atmospheric Deposition of current use pesticides in the Arctic: Snow core records from the Devon Island Ice Cap, Nunavut, Canada.” *Environmental Science: Processes & Impacts* 2013(15): 2304–2311.
